# Ultrafast investigation of photoinduced charge transfer in aminoanthraquinone pharmaceutical product

**DOI:** 10.1038/srep43419

**Published:** 2017-02-24

**Authors:** Song Zhang, Simei Sun, Miaomiao Zhou, Lian Wang, Bing Zhang

**Affiliations:** 1 State Key Laboratory of Magnetic Resonance and Atomic and Molecular Physics, Wuhan Institute of Physics and Mathematics, Chinese Academy of Sciences, Wuhan, Hubei 430071, China

## Abstract

We investigated the mechanism of intramolecular charge transfer and the following radiationless dynamics of the excited states of 1-aminoanthraquinone using steady state and time-resolved absorption spectroscopy combined with quantum chemical calculations. Following photoexcitation with 460 nm, conformational relaxation via twisting of the amino group, charge transfer and the intersystem crossing (ISC) processes have been established to be the major relaxation pathways responsible for the ultrafast nonradiative of the excited S_1_ state. Intramolecular proton transfer, which could be induced by intramolecular hydrogen bonding is inspected and excluded. Time-dependent density functional theory (TDDFT) calculations reveal the change of the dipole moments of the S_0_ and S_1_ states along the twisted coordinate of the amino group, indicating the mechanism of twisted intra-molecular charge transfer (TICT). The timescale of TICT is measured to be 5 ps due to the conformational relaxation and a barrier on the S_1_ potential surface. The ISC from the S_1_ state to the triplet manifold is a main deactivation pathway with the decay time of 28 ps. Our results observed here have yield a physically intuitive and complete picture of the photoinduced charge transfer and radiationless dynamics in anthraquinone pharmaceutial products.

Over the past decade there has been a considerable amount of research toward understanding the unimolecular deactivation pathway of photoexcited pharmaceutical products in the presence of biological substrates^1^,^2^,^3^,^4^,^5^. Anthraquinone (AQ) and its derivatives are a constituent of the chromophore of hypericin, which is naturally occurring in St. John’s wort and other plants of the Hypericum genus. Hypericin is used as a photosensitizer in photodynamical therapy of cancer and displays photoinduced virucidal (anti-viral, anti-retroviral) and antitumor activities^6^,^7^,^8^,^9^,^10^. Moreover, when anthraquinone is designed as photonucleases and reacts with DNA, they can cleave the single or double-strand DNA at special sites^11^,^12^,^13^,^14^. J. R. Wagner and his co-worker reported the formation of novel interstrand corss-links between anthraquinone and thymine in DNA^13^. D. Zhong and A. H. Zewail *et al*. discussed femtosecond primary dynamics for anthracycline antibiotics function^15^.

Quinones are also widely employed as electron and hydrogen atom acceptors in thermal and photochemical processes by nature as well as in man-made systems^16^,^17^,^18^. According to picosecond laser spectroscopy, it is considered that the excited quinones react with DNA by two separate oxidative pathways: hydrogen atom abstraction from the deoxyribose component of the nucleic acid backbone and electron transfer from a nearby base^19^. Hydroxyl and amino substitution of anthraquinone has a pronounced effect on its electronic structure and photophysical properties. The mechanism of intramolecular hydrogen-bonded and the dynamics of intramolecular proton-transfer have been revealed by time-resolved fluorescence and absorption spectroscopy. Ryu and S. Sun *et al*. determined that excited-state intramolecular proton transfer (ESIPT) of 1- hydroxyanthraquinone is less than 50 fs using fluorescence up-conversion by two-photon excitation and femtosecond transient absorption spectroscopy, respectively^20^,^21^. Ammonium-substituted AQ derivatives have been found to catalyze DNA cleavage upon irradiation with 350 nm light^19^,^22^,^23^,^24^. Aminoanthraquinone is a simplest AQ derivative and has two chromophoric groups, namely AQ and the amino groups. However, 1-aminoanthraquinone, as well as 1, 8-dihydroxyanthraquinone and 9-hydroxyphenalenone are considered to be intramolecular hydrogen-bonded model systems. Of special interest are the derivatives with strong intramolecular hydrogen bonds to the quinone oxygen. A dominant hydrogen-bonding interaction between the carbonyl oxygen with solvent might have a distinctive influence on the fluorescence behaviour. Fluorescence quantum yields were measured to be 0.006~0.0715 in different solvents by steady absorption and time-resolved spectra^25^,^26^. In addition, the nonradiative relaxation mechanism is attributed to internal conversion to the S_0_ state. However, the lowest excited states of aminoanthraquinones have intramolecular charge transfer characters. It cannot be negligble that Wasielewski pointed out that AQ derivatives undergo rapid intersystem crossing to yield relatively long-lived triplet states that are capable of oxidizing purine nucleobases^27^.

In this paper, we report on a joint experimental and calculational study of the excited-state dynamics of 1-aminoanthraquinone in solution after excitation to the charge transfer electronic state. We employ femtosecond transient absorption spectroscopy to monitor the temporal evolution of the photoexcited 1-aminoanthraquinone in solution and elucidate unimolecular deactivation pathway of, especially twisted intramolecular charge transfer and following ISC processes. The characteristic spectra bands were measured and analyzed in detail combined with quantum chemical calculations. The kinetic traces of transient absorption disclose a mechanism of geometry relaxation and contribution of the triplet states. Quantum chemical calculations are also performed to help to understand the suggested mechanism.

## Results and Discussion

### Steady and transient absorption spectra

**Figure 1:**
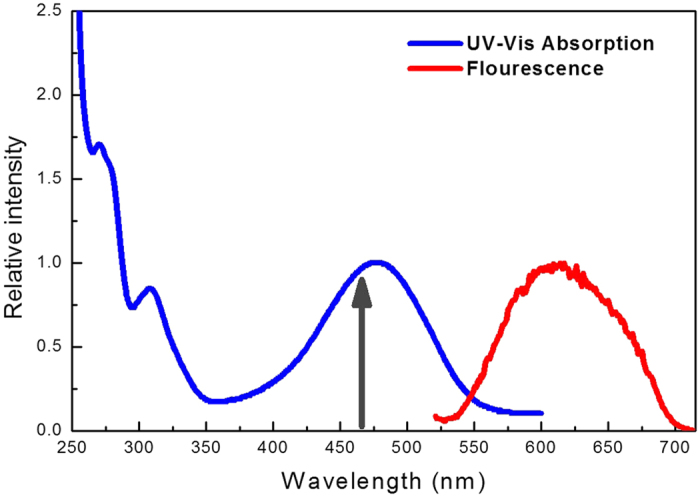
Steady absorption and fluorescence spectra with the excitation at 460 nm of 1-NH_2_-AQ in ethanol.

The absorption and fluorescence spectra of 1-NH_2_-AQ in ethanol are shown in Fig. 1. As can be seen, the absorption spectrum at wavelength of λ > 250 nm reveals several distinguishable absorption bands. These bands correspond to the transitions from the ground state to the different excited states. A relative much broader absorption band is peaked at 480 nm and associated with a transition from the S_0_ state to the first optically bright S_1_ state, which has a large oscillator strength. Following an excitation at 460 nm to the S_1_ state, a redshifted and broad emission is observed. An enormously large Stokes shifted band with respect to the S_0_-S_1_ absorption band is located at 620 nm in the fluorescence spectra. The emission with such a large Stokes shift indicates that the molecule has undergone a significant rearrangement.

**Figure 2:**
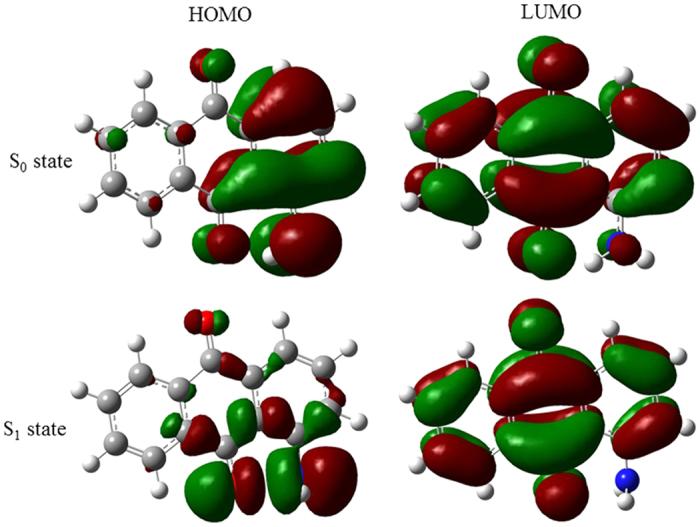
Molecular orbits of HOMO and LUMO at the optimized S_0_ and S_1_ structures using (TD) DFT/B3LYP/6-311G, respectively. The orbital wave functions are positive in the red regions and negative in the green.

All optimized geometries are determined to be planar structures with *C*_*s*_ symmetry on B3LYP method with 6-311G basis set and confirmed to be stationary points by vibrational frequencies analysis. The chromophoric groups reside on the same plane in the structure of the ground state. Jürgen Troe *et al*. also confirmed that the equilibrium conformation is almost complete planarity on the HF/6-31G(d,p)^28^. All calculations under the planar structure reveal that the lowest excited S_1_ state is a bright state and originates a 59 ← 58 transition, whereas the second excited S_2_ state mainly originates a 59 ← 57 transition and is a dark state for its zero transition dipole moment. The molecular orbits of HOMO and LUMO at optimized S_0_ and S_1_ structures are shown in Fig. 2, respectively. The 57 and 58 orbits are both π orbits and correspond to the HOMO-1 and HOMO, respectively, whereas the 59 orbit is the LUMO and belongs to a π* orbit. The calculations show that the excitation to the S_1_ state is strongly allowed with the main contribution of the transition from the HOMO to the LUMO. For the optimized geometry of the S_0_ state, the HOMO is largely localized on the amino group, whereas the LUMO is largely localized at the whole anthraquinone group. The calculated vertical excitation energies of the excited states at a planar form using several methods with different basis sets are listed in Table 1. The original transition to the first populated excited state of 1-NH_2_-AQ was determined to be 2.6325 eV using fluorescence excitation spectra^28^. The calculated vertical excitation energies of the S_1_ state are good match with the original transition to the S_1_ state. The value at B3LYP/6-311G level is the most consistent with the experimental value.

Since the B3LYP/6-311G level gives the minimum energy of the ground state and the most consistent energy value of the S_1_ state, the other calculations in this work are performed at the same method level.

**Figure 3:**
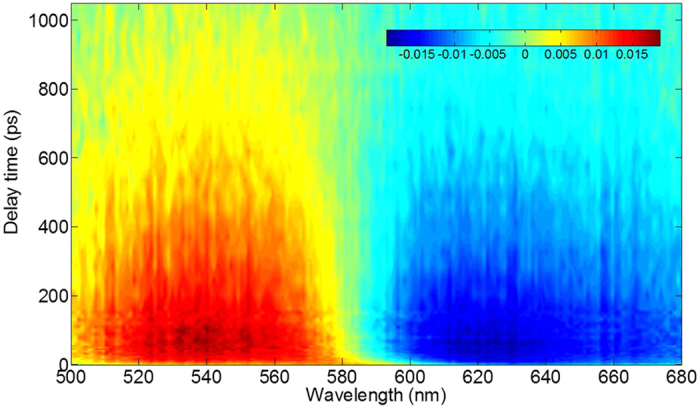
Transient absorption spectra of 1-NH_2_-AQ in ethanol. The color intensity reflects signal magnitude. As indicated by the color scale bars, the red and blue represent the excited state absorption (ESA) and simulated emission (SE), respectively.

**Figure 4:**
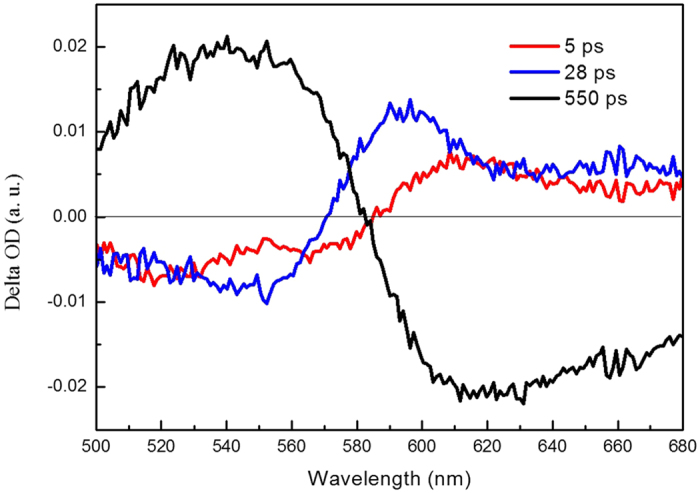
Decay-associated difference spectra (DADS) by global fit analysis.

**Figure 5:**
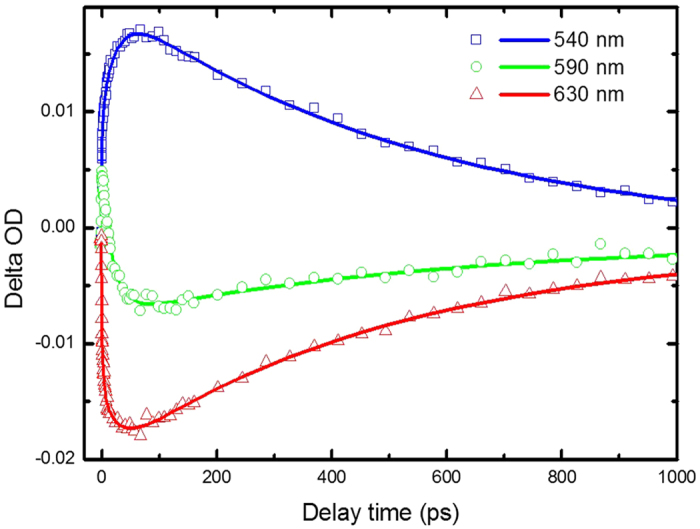
Kinetic traces of the transient absorption spectra at several probe wavelengths. The symbols are data, solid lines the overall least-squares fit curves.

The pump wavelength is 460 nm and coincides with the peak of the ππ* absorption band of the molecules. The transient absorption spectra of 1-NH_2_-AQ in ethanol are measured from 500 to 680 nm, as shown in Fig. 3. It obviously divides into two main broad bands. The region at wavelength < 590 nm is a positive signal and elucidated by the excited state absorption (ESA). And the other region from 590 to 680 nm is a negative signal and originating from the stimulated emission (SE). The two main bands become weaker as the pump-probe delay time increases. Both of them almost disappear until 900 ps. To properly describe the dynamics observed in the visible range, we performed a singular value decomposition (SVD) analysis on the 2D data matrix. The resulting kinetic amplitude vectors were globally fitted. Three time-constants of 5 ps, 28 ps, and 550 ps determined by global fit results are listed in Table 2. The decay associated difference spectra (DADS) shown in Fig. 4. do overlap, indicating that the temporal development of the spectral features cannot be described with a single exponential function. The DADS reflects the relative spectral contributions of each time-component. The global analysis result shows a good match with the experimental traces over the whole spectro-temporal range, and the fit parameter values are in good agreement with the parameters from the SVD analysis. Time profiles of the transient absorption at several different wavelengths of 1-NH_2_-AQ in ethanol are shown in Fig. 5. Several typical wavelengths of 540, 590 and 630 nm are chosen to represent the temporal behavior of ESA and SE bands, respectively. Over the whole spectro-temporal range, the experimental time profiles can be described with a first exponential with several picoseconds decay time, τ_1_ = 5 ps and rather long-lived exponentials, τ_2_ = 28 ps and τ_3_ = 550 ps, respectively. Roughly, the amplitudes of three components in ESA band are opposite to those in SE band respectively, and the weights of the component τ_3_ in both bands are the main contribution since their amplitudes are obviously larger than others.

### Intramolecular hydrogen bonding

Intramolecular hydrogen bonding and dynamics of intramolecular proton transfer (PT) have been investigated in three intramolecular hydrogen-bonded molecules, 1, 8dihydroxyanthraquinone (1,8-DHAQ), 1-NH_2_-AQ, and 9-hydroxyphenalenone (9-HPA), respectively^28^. According to our calculations, the distance between O atom and adjacent H atom in NH_2_ group of 1-NH_2_-AQ is only 1.88 Å. It is close to the distance between O atom and adjacent H atom in OH group of 1-HAQ, which is calculated to be 1.72 Å under the same calculation level. S. Sun *et al*. mentioned that the ESIPT of 1-HAQ is as fast as only ~32 fs^20^. The original transition to the first populated excited state of jet-cooled 1-HAQ was determined to be at 461.98 nm using laser induced fluorescence and resonant 1 + 1’ ionization^29^. It is noticed that the O-H stretching vibration is at 2940 cm^−1^ to the red of the original band in the emission spectra and can be directly excited by the 400 nm pump pulse in Franck-Condon region. The directly exited O-H stretching mode is devoted to ultrafast ESIPT. However, for 1-NH_2_-AQ, the original transition to the first excited state is 471 nm in gas, 497 nm in heptane and 542 nm in ethanol, respectively^28^,^30^,^31^. The vibrational spectrum of the ground state are calculated on B3LYP/6-311G level. It determined that the N-H stretching vibrations are 3211 and 3312 cm^−1^, which are assigned symmetry and as-symmetry vibrational modes, respectively. The excitation with 460 nm is just 3289 cm^−1^ above the original band of the S_1_ state in ethanol. Considering bandwidth, it is obviously that the both N-H stretching modes are directly excited and promote IHB in some certain extent. G. D. Gllllsple *et al*. mentioned that it is well-known for A-H···B hydrogen-bonded systems, the stronger the H···B hydrogen bond, the lower the A-H stretching frequency^31^. For hydroxyl aromatics, O-H stretching frequency is 3600 cm^−1^ in non-hydrogen-bonded system. It is interesting that O-H stretching decreases to 2940 cm^−1^ in 1-HAQ. Moreover, two characteristic N-H stretching modes near 3400 and 3500 cm^−1^ exist in amino-substituted aromatic molecules in which there is no IHB. For 1-NH_2_-AQ, N-H stretching are 3332 and 3502 cm^−1^ measured by Zaitsev and 3211 and 3312 cm^−1^ in ethanol calculated on B3LYP/6-311G level, respectively. IHB is similar with those in 2-NH_2_-AQ and 2-aminoanthracene. It can be obviously concluded that the H atom of N-H bonds are involved in weaker intramolecular hydrogen bonds than the H atom of OH bonds. Furthermore, Müller *et al*. suggested that 1-NH_2_-AQ are associated with a single-minimum-type potential^28^. They checked the presence of a HT barrier along the reaction path calculated with CIS method with electron correlation by TDDFT. According to the TDDFT calculations, the HT barrier in 1-NH_2_-AQ is 3300 cm^−1^. The excess energy in the S_1_ state following the excitation with 460 nm is obviously lower than the barrier. It is disadvantage for ESIPT due to the difficulty to pass through the higher barrier.

### Intramolecular charge transfer

Optimized structures of the ground and first excited states of 1-NH_2_-AQ are shown in Fig. 2, respectively. It is interesting that the optimized geometry of the S_1_ state is no longer a planar, but a twisted. The amino group twists and is almost perpendicular to the anthraquinone plane. The dihedral angle between the NH_2_ group and anthraquinone at the optimized structure of the S_1_ state becomes about 90°. Furthermore, the excited state optimized geometrical parameters reveal an interesting feature that the C=O bond nearby the amino group and the C-N bond increase from 1.270 and 1.363 Å in the S_0_ state to 1.321 and 1.442 Å in the S_1_ state, respectively. It originates from the strong donating nature of the amino group, which induces a large charge localization at the amino group.

The dipole moments of the S_0_ and S_1_ states are determined to be 2.3818 D and 7.8076 D based on B3LYP/6-311G, respectively. The dipole moment of the excited state, in which the -NH_2_ group is twisted, is obviously larger than that having the coplanar conformation. A change of the transition dipole moment of ~5.4258 D manifests a charge transfer process in molecule upon an excitation. The electron cloud distributions of both conformations also show the transfer of the charge. It is also apparent from the HOMO-LUMO electron distribution in the ground and the excited state geometries. As shown in Fig. 2, in the optimized geometry of the S_1_ state, the LUMO electron density is more largely localized at the anthraquinone group as compared to that in the S_0_ state. It is obvious that the LUMO electron has been pulled from electron-donating –NH_2_ group to electron-withdrawing anthraquinone group. The electron density distributions suggest that the S_1_ state is a strong ICT character.

As mentioned above, the conformation of 1-NH_2_-AQ maintains a coplanar structure in Franck-Condon region after excitation and relaxes to a twisted structure on the potential surface of the S_1_ state. The parameters including the energies and dipole moments of the S_0_ and S_1_ states as a function of the twisted angle between the amino and anthraquinone groups are calculated using B3LYP method with 6-311G basis set and listed in Table 3. It is noticed that the oscillator strengths are decreasing as the twisted angle increasing. The oscillator strength becomes zero at 90° of the angle. It is obviously suggested that the fluorescence just emissions from an initially locally excited state not via a relaxed ICT state. No dual fluorescence was also observed in other ICT molecular system^32^,^33^,^34^. The dipole moments of the S_0_ state is decreasing as the twisted angle increasing, whereas the dipole moments of the S_1_ state has the opposite trend. The dipole moment of the planar conformation in the S_0_ state is 2.3818 D. Following excitation to the Franck-Condon region, the dipole moment suddenly increases to 6.7567 D. Furthermore, the dipole moment of the S_1_ state increases to 7.8076 D with increasing of the twisted angle between the amino group and the anthraquinone moiety. It is elucidated that the twisted intramolecular charge transfer is associated with the conformational relaxation on the potential surface of the S_1_ state. It is also proved by the decrease of the charge localized at N atom from 0.906 e^−^ in the S_0_ state to 0.650 e^−^ in the S_1_ state. However, the timescale of twisted intra-molecular charge transfer (TICT) is usually measured to be a few hundred femtoseconds in some barrierless systems^32^,^33^,^34^. It is considered the relative energies of the S_1_ state to the ground state at different -NH_2_ twisted angles to properly depict the potential. For 1-NH_2_-AQ, the energy at the 0° twisted angle in the S_1_ state is just 2.6756 eV, whereas it becomes 2.9351 eV at the 60° twisted angle. It indicates that there exists a small barrier about 0.2595 eV on the potential surface of the S_1_ state along the twisted coordinate of the amino group. Müller *et al*. also suggested that a single-minimum-type potential exists in the S_1_ states of 1-NH_2_-AQ^28^. The TICT process in 1-NH_2_-AQ is estimate to be longer since the small barrier on the amino twisting potential surface exists. The fast decay component of 5 ps is definitely attributed to twisted intramolecular charge transfer.

**Figure 6:**
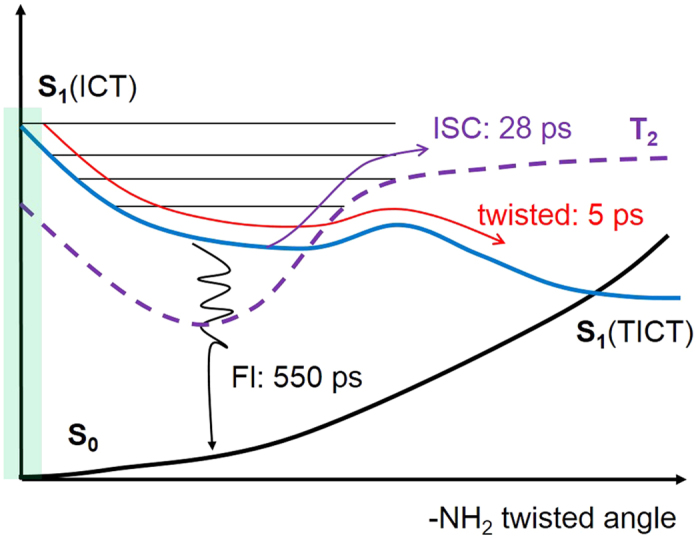
Schematic diagram of electronic energies and proposed radiationless dynamics of the excited states of 1-NH2-AQ. ICT, intramolecular charge transfer; TICT, twisted intramolecular charge transfer; ISC, intersystem cross; FL, Fluorescence.

As mentioned in Table 3, it is obvious that the energies of the S_0_ and T_2_ states are increasing as the twisted angle increasing, whereas the energies of the S_1_ and T_1_ states are decreasing. It is estimated that the potential energy surfaces of the S_1_ and T_2_ states become isoenergetic nearby the twisted angle of ~40°. These calculations also govern the presence of a prominent conical intersection between the S_1_ and T_2_ states. It is possible that the deactivation of the S_1_ state is directed to the intersystem crossing (ISC) channel. In the case of 1-NH_2_-AQ, the intersystem crossing to the triplet state is a major deactivation channel from the S_1_ state and in this derivative a close-lying T_2_ state seems to be responsible for the high k_isc_ rate. The second time component of 28 ps was assigned to the ISC from the S_1_ state to the triplet manifold. The potential surface of the ground, triplet and singlet excited states. of 1-NH_2_-AQ along as the change of twisted angle using B3LYP/6-311G are plotted in Fig. 6. Moreover, the energy gap between the S_1_ and S_0_ states in Franck-Condon region is 2.6756 eV. However, the energies of the S_0_ and S_1_ states at the optimized geometry of the S_1_ state were performed at the same method level and determined to be 1.6699 eV and 2.2748 eV, respectively. The energy gap of both states is only 0.6049 eV at the optimized geometry of the S_1_ state. It can be deduced that the energy gap decreases from 2.6756 eV to 0.6049 eV along the conformation relaxation coordinate. The obviously decrease trend of the energy gap is benefited to an ultrafast internal conversion (IC) from the S_1_ state to the S_0_ state. Venkataraman *et al*. pointed out that the S_1_ state is mainly deactivated through IC to the ground state. The rate constant of IC is determined to be 5.3 × 10^8^ and 2.3 × 10^9^ s^−1^ in toluene and methanol, respectively^25^. It is obvious that the rate constant of IC is much larger than that of ISC. Yoshihara *et al*. pointed out that the absorption and fluorescence spectra is dependence on the strong solvent polarity and the lifetime of S_1_ state is about 400 ± 100 ps in ethanol by picosecond fluorescence studies^26^. It agreed well with the value of τ_3_ = 550 ps obtained in our measurements. A longer decay component is observed in ethanol and assigned to be the lifetime of the S_1_ state.

## Conclusions

In this paper, we inspected the mechanism of intramolecular charge transfer and following radiationless dynamics of the excited states of 1-NH_2_-AQ using time-resolved absorption spectroscopy combined with quantum chemical calculations. Two main absorption bands were illustrated by the excitation at 460 nm to the S_1_ state. The involvement of -NH_2_ group rotation become as the main coordinate in the excited state relaxation dynamics. The optimized structure of the ground state is confirmed to be a planar conformation with *C*_*s*_ symmetry, whereas the structure of the S_1_ state is a twisted conformation with the amino group perpendicular to the anthraquinone plane. The difference of the dipole moments of the S_0_ and S_1_ states is ~5.4258 D and manifests a charge transfer process in molecule upon an excitation. The increase of dipole moment in the S_1_ state with the change of the twisted angle is elucidated that the TICT is associated with the conformational relaxation on the potential surface of the S_1_ state. The fast decay component of 5 ps is definitely attributed to twisted intramolecular charge transfer. Afterwards, the ISC from the S_1_ state to the triplet manifold is a main deactivation pathway with the decay time of 28 ps. The long-lived triplet state plays the role of oxidizing purine nucleobases. According to the ES band and the larger rate constant of IC, the population of the S_1_ state is decayed by way of fluorescence. A general photoinduced mechanism is drawn in Fig. 6 according to the experiments and quantum chemical calculations.

## Experimental Method

1-aminoanthraquinone (1-NH_2_-AQ, 99% purity) was purchased from Sigma, and used without further purification. Ethanol (99% purity) purchasing from Aladdin was used as a solvent. The concentration of 1-NH_2_-AQ in ethanol was 1 mM at room temperature and a fresh sample was prepared for each measurement. The absorption and emission spectra were recorded on the UV-VIS spectrometer (INESA, L6) and the spectrometer (Princeton, SpectraPro 2500i) in a 1 mm quartz cell, respectively.

Ultrafast broadband absorption measurements were performed based on a Ti:sapphire femtosecond laser system. Details of the femtosecond laser system have been described elsewhere^35^,^36^. Briefly, the seed beam is generated by a commercial Ti:sapphire oscillator pumped by a CW second harmonic of an Nd:YVO_4_ laser, and then amplified by an Nd:YLF pumped regenerative amplifier to generate a 1 kHz pulse train centered at 800 nm of approximately 35 fs pulse width and with maximum energy of 1 mJ/pulse. A fraction of the laser is frequency doubled in a 1 mm thick BBO crystal, yielding pulses at 400 nm with an energy of 100 μJ, which are used to pump the NOPA. The excitation pulse energy at 460 nm used here is about 2 μJ by an attenuation. The NOPA pulse needs to be temporally compressed in order to obtain the minimum pulse width compatible with their bandwidth. A white light continuum generated by focusing the fundamental light at 800 nm on a 1 mm sapphire plate is reflected from the front and back surfaces of a quartz plate to obtain the probe and reference beams. The pump and probe pulses intersect in the sample at an angle of ~ 4°, and the reference beam is transmitted through the sample at a different spot. The relative polarization of the pump and probe pulses is set to the magic angle for all the measurements. A linear translation stage is used to delay the probe beam to monitor the pump-probe dynamics. The resulting spectra are detected by a CCD camera (PI-MAX, 1024 × 256 pixel array) equipped with a spectrometer (Princeton, SpectraPro 2500i). The instrumental response function of the system, determined by cross correlation between the excitation and probe pulses using the optical Kerr-gate method, is typically better than 150 fs.

All quantum chemical calculations are performed using the Gaussian09W suit of program^37^. The geometries of the ground and excited states of 1-NH_2_-AQ are optimized using MP2 and B3LYP with 6-311G basis set in gas phase and ethanol solution, respectively. The stationary points are also confirmed by the vibrational frequencies analysis. The energies of excited states are performed using the B3LYP function based on optimized geometries of the ground and excited states, respectively. The B3LYP function provides accurate excited-state ordering, excited-state transition energies, oscillator strengths, transition dipole moments and singlet-triplet energy gaps, particularly when solvent effects are taken into account^38^,^39^, which has been performed in other molecular systems^32^,^34^,^40^,^41^. Solvent effects are expected to lead to large ground- and excited-state energy changes in heteroaromatic compounds. Thus, the effect of the bulk solvent dielectric on the ground-state geometries and on the excited-state vertical energies was modeled by performing self-consistent reaction field (SCRF) calculations using the polarizable continuum model (PCM) with the integral quation formalism^42^,^43^.

## Additional Information

**How to cite this article:** Zhang, S. *et al*. Ultrafast investigation of photoinduced charge transfer in aminoanthraquinone pharmaceutical product. *Sci. Rep.* **7**, 43419; doi: 10.1038/srep43419 (2017).

**Publisher's note:** Springer Nature remains neutral with regard to jurisdictional claims in published maps and institutional affiliations.

## Figures and Tables

**Table 1:** Vertical excitation energies (eV) and oscillator strengths (*f*) of 1-NH_2_-AQ at the optimized geometry of the S_0_ state with B3LYP/6-311G.

State	Orbit	B3LYP/6-311G	B3LYP/lanl2dz	B3PW91/6-311G	B3PW91/lanl2dz
S_1_	58–59	2.6757	2.6849	2.6825	2.6970
		*f* = 0.1239	*f* = 0.1260	*f* = 0.1249	*f* = 0.1278
S_2_	57–59	2.8895	2.8350	2.8732	2.8169
		*f* = 0	*f* = 0	*f* = 0	*f* = 0

**Table 2:** Results of the global fit analysis of the absorption–time profiles of 1-NH_2_-AQ in ethanol.

λ(nm)	a_1_	τ_1_(ps)	a_2_	τ_2_(ps)	a_3_	τ_3_(ps)
520	−0.044(1)	5.0(2)	−0.045(1)	28(3)	0.127(1)	550(6)
540	−0.034(1)	5.0(2)	−0.059(1)	28(3)	0.144(1)	550(6)
560	−0.027(1)	5.0(2)	−0.050(1)	28(3)	0.133(1)	550(6)
580	−0.008(1)	5.0(2)	0.046(1)	28(3)	0.023(1)	550(6)
590	0.017(1)	5.0(2)	0.080(1)	28(3)	−0.054(1)	550(6)
600	0.040(1)	5.0(2)	0.074(1)	28(3)	−0.107(1)	550(6)
632	0.039(1)	5.0(2)	0.032(1)	28(3)	−0.130(1)	550(6)
652	0.029(1)	5.0(2)	0.034(1)	28(3)	−0.109(1)	550(6)
672	0.029(1)	5.0(2)	0.033(1)	28(3)	−0.096(1)	550(6)

Values in parentheses give the 2σ standard deviations with respect to the last digits.

**Table 3:** The ground state energies, vertical excitation energies, oscillator strengths (*f*), dipole moments (μ) of the S_0_ and S_1_ states at the optimized S_0_ and S_1_ structures of 1-NH_2_-AQ along as the change of twisted angle by using (TD)DFT/B3LYP/6-311G, respectively. S and T represent the singlet and triplet excited states, respectively.

Angle (°)	S_0_ (eV)	T_1_ (eV)	T_2_ (eV)	S_1_ (eV)	*f* (S_1_)	μ(S_0_) (D)	μ(S_1_) (D)
0	0	1.8248	2.4579	2.6756	0.1239	2.3818	6.7567
10	0.03473	1.8322	2.4571	2.6600	0.1130	2.3713	6.5601
20	0.13278	1.8478	2.4544	2.6113	0.0891	2.3379	6.2137
30	0.27807	1.8588	2.4506	2.5285	0.0652	2.1713	6.0040
40	0.45092	1.8432	2.4538	2.4163	0.0465	2.1564	5.9470
50	0.63300	1.7910	2.4776	2.2814	0.0325	1.9825	5.9968
60	0.80787	1.7084	2.5256	2.1272	0.0214	1.7508	6.1831
70	0.95866	1.6120	2.5411	1.9567	0.0117	1.4849	6.6126
80	1.06453	1.5266	2.5346	1.7952	0.0036	1.2459	7.3332
90	1.10331	1.4903	2.5318	1.7205	0	1.1434	7.8076
